# Physical activity and mental health in children and youth during COVID-19: a systematic review and meta-analysis

**DOI:** 10.1186/s13034-023-00629-4

**Published:** 2023-07-19

**Authors:** Bowen Li, Kwok Ng, Xiuhong Tong, Xiao Zhou, Jiangchuan Ye, Jane Jie Yu

**Affiliations:** 1 Department of Sport and Exercise Science, College of Education, Zhejiang University, Hangzhou, China; 2 Physical Activity for Health Research Cluster, Department of Physical Education and Sport Sciences, University of Limerick, Limerick, Ireland; 3 Faculty of Education, University of Turku, Rauma, Finland; 4 School of Educational Sciences and Psychology, University of Eastern Finland, Joensuu, Finland; 5 Department of Psychology, The Education University of Hong Kong, Hong Kong, China; 6 Department of Psychology and Behavioral Sciences, Zhejiang University, Hangzhou, China

**Keywords:** SARS CoV-2, Depression, Anxiety, Stress, Well-being, Positive psychology

## Abstract

**Background:**

The coronavirus disease (COVID‐19) and universal mitigation strategies have fundamentally affected peoples’ lives worldwide, particularly during the first two years of the pandemic. Reductions in physical activity (PA) and increased mental health (MH) problems among children and youth have been observed. This systematic review and meta-analysis investigated the relationship between physical activity (PA) and mental health (MH) among children and youth during the COVID‐19 pandemic.

**Methods:**

Four electronic databases (EMBASE, PsycINFO, PubMed, and Web of Science) were systematically searched to identify studies that (1) examined the relationship between PA and MH among children and youth (aged 2–24 years old) and (2) were published in peer-reviewed journals in English between January 2020 and December 2021. Relationships between PA and two MH aspects (i.e., negative and positive psychological responses) among children and youth at different age ranges and those with disabilities or chronic conditions (DCC) were synthesized. Meta-analyses were also performed for eligible studies to determine the pooled effect size.

**Results:**

A total of 58 studies were eventually included for variable categorization, with 32 eligible for meta-analyses. Our synthesis results showed that greater PA participation was strongly related to lower negative psychological responses (i.e., anxiety, depression, stress, insomnia, fatigue, and mental health problems) and higher positive psychological responses (i.e., general well-being and vigor) in children and youth during COVID-19. The pattern and strength of relations between PA and MH outcomes varied across age ranges and health conditions, with preschoolers and those with DCC receiving less attention in the existing research. Meta-analysis results showed that the magnitude of associations of PA with negative (Fisher’s z = − 0.198, *p* < 0.001) and positive (Fisher’s z = 0.170, *p* < 0.001) psychological responses among children and youth was weak. These results were linked to age of participants, study quality, and reporting of PA-related information.

**Conclusions:**

PA participation and MH among children and youth deteriorated during the COVID-19 pandemic and were closely associated with each other. For the post-COVID-19 era, additional research on age- and health condition-specific relationships between PA and MH outcomes from a comprehensive perspective is warranted. (Word count: 344 words).

**Supplementary Information:**

The online version contains supplementary material available at 10.1186/s13034-023-00629-4.

## Introduction

Mental Health (MH) problems constitute the largest health concern for children and youth worldwide in the twenty-first century [1]. Representing MH states are two psychological responses: negative, which consist of unpleasant feelings or emotions and symptoms related to clinically diagnosed ill-being [2, 3]; and positive, which consist of affective states and psychological well-being beneficial to one’s life [2, 4–8]. MH is influenced by many factors [9], including physical activity (PA), referred to as any type of physical movement that increases energy expenditure [10]. It has been documented that regular PA participation is protective for MH by preventing and managing negative psychological responses [10, 11]. Engaging in an adequate level of PA regularly is important for promoting people’s physical and mental health, especially for children and youth [12]. However, the majority of young people are not meeting WHO’s PA guideline (i.e., an average of 60 min per day of moderate-to-vigorous PA intensity), which has resulted in a worldwide critical public health issue [13]. Moreover, a number of studies have demonstrated that physical inactivity deteriorates as age increases [14, 15].

PA and MH are theoretically and empirically associated, and the relation is bidirectional [16]. For example, Sampasa-Kanyinga [17] reported that a low level of PA is closely related to high levels of negative psychosocial responses (e.g., anxiety, depression, stress, negative affect, and distress) among children and youth. Not surprisingly, other studies found that a high level of PA is related to positive outcomes, such as well-being, self-esteem, self-concept, and resilience [16–18]. As early as 2011, a meta-analysis based on randomized controlled trials (RCTs) revealed that increased levels of PA are significantly associated with improved MH among children [19]. In 2019, another meta-analysis reported that the effects of PA on psychological ill-being (effect size = 0.130, *p* = 0.007) and psychological well-being (effect size = 0.189, *p* = 0.001) among children and youth are small but significant [16]. Thus, as improvements in MH reflect fewer negative psychological responses and more positive ones [20, 21], both positive and negative psychological responses should be considered when facilitating a comprehensive understanding of the relation between PA and MH.

The coronavirus disease (COVID-19) is an ongoing pandemic caused by a novel coronavirus, the severe acute respiratory syndrome coronavirus-2 (SARS-CoV-2) [22]. Many countries implemented physical distancing measures, national lockdowns, and travel restrictions to control the spread of COVID-19 during the first two years of the outbreak [23]. As a protection for children and youth, restrictions to physically attend a majority of schools and universities were implemented worldwide, with an estimated 1.5 billion students transitioning to online learning [24]. These school restrictions and other social behavioral adaptations (e.g., social distancing, isolation) severely impacted the 24 h lifestyle of many children and youth [25], resulting in decreased opportunities for PA and increased sedentary behavior [26].

Moreover, the outbreak of COVID-19 has been accompanied by significant global MH challenges [27]. For example, an increasing number of studies have reported higher levels of anxiety, depression, and stress among children (ages 6–12 years) who experienced family isolation and school closures during COVID-19 [28–30]. Other studies have shown that people who were able to maintain more total time in moderate to vigorous PA were 12–32% less likely to experience depressive symptoms and 15–34% less likely to experience anxiety during COVID-19 [28, 31–33]. Although several existing reviews published in 2021 and 2022 demonstrated the relation between PA and MH among children during COVID-19 [28, 31, 33–36], they focused mainly on specific negative psychological responses (i.e., anxiety, depression and stress) with only a limited number of eligible studies being included. Additionally, the impacts of COVID-19 on PA and MH appear to be greater among children and youth with disabilities or chronic conditions (DCC) than their peers without DCC [28, 37]. However, within the COVID-19 context, a comprehensive understanding of the relationship between PA and MH among children and youth, including those with DCC, is still lacking. The relationship between PA and MH may be stronger in children and youth during COVID-19 than before COVID-19; and the pattern and extent of this relationship may vary by age range and disability status.

According to the theory of positive psychology, an adversity can enhance the ability of certain populations to cope positively and creatively with stress and distress [38]. During the COVID-19 pandemic, some people with an optimistic mindset counteracted negative impacts by making the most of limited resources and by being physically active and exercising at home [38, 39]. It is important to encourage children and youth to overcome the negative impacts of the COVID-19 pandemic by using methods, such as PA participation, that foster positive emotions and optimism [40]. Therefore, the purpose of this systematic review and meta-analysis was to evaluate the association of PA with MH in terms of both negative and positive psychological responses among children and youth within the context of COVID-19. Specifically, we asked two questions: (1) Did close relationships between PA and MH outcomes occur among children and youth during COVID-19?; and (2) Did the pattern and magnitude of such relationships vary by age range and disability status? We hypothesized that: (1) there would be close relationships between PA and MH outcomes in terms of both negative and positive psychological responses among children and youth during COVID-19; and (2) the pattern and magnitude of the relationships would vary in different age ranges and health conditions (i.e., with and without DCC). Information gained in this systematic review and meta-analysis will not only facilitate a better understanding of the relation between PA and MH, but also inform new research on how to promote PA and MH among children and youth during any future COVID-19-type pandemic.

## Method

The conduct and reporting of this review followed the Preferred Reporting Items for Systematic Reviews and Meta-Analysis (PRISMA) guidelines [41]. Registration for this protocol was completed on the Prospero database (reference number: CRD42022303342).

### Search strategy

Four electronic databases (i.e., Embase, PsycINFO, PubMed, and Web of Science) were systematically searched to identify relevant studies published between January 2020 and December 2021. This end date was chosen since the impact of COVID-19 on daily life, including isolation policies, gradually diminished in many countries starting in the second half of 2021 [42–45]. The search items were grouped into four components: (1) physical activity (physical activity OR exercise OR sport), (2) mental health (mental health OR mental problem OR mental illness OR mental disorder OR well-being OR depression OR anxiety OR stress OR happiness), (3) age group of interest (child OR adolescent OR youth), and (4) COVID-19 (COVID-19 OR SARS-CoV-2). An example of the search strategy can be found in Additional file 1: Table S1.

### Eligibility criteria

Inclusion criteria were: (1) participants were children and youth aged 2 to 24 years with and without DCC; (2) studies reported a potential relationship between any type of PA and at least one MH outcome in the context of COVID-19, which enabled evidence for specific correlates to be determined; (3) studies used a cross-sectional, longitudinal, or experimental design; and (4) articles were published in a peer-reviewed journal in English. Qualitative studies, reviews, books, dissertations, conference proceedings, commentaries, and studies without full-text were excluded.

### Study selection

After conducting the initial search and removing all duplicates, two reviewers (BL, JY) were trained to independently perform title/abstract and full-text screening for the inclusion of records with a *yes*, *unsure*, or *no* approach. Inter-rater reliability between the two reviewers at the two screening phases was calculated using the κ statistic. κ values between 0.60 and 0.74 were deemed as having good agreement, and values > 0.75 were deemed as having excellent agreement [46]. Any discrepancy between the two reviewers at any stage was jointly reviewed and discussed with a third reviewer (JJY) until a consensus was achieved.

### Quality assessment

Two reviewers (BL, JY) independently rated the methodological quality of all the included studies. Inter-rater reliability was calculated using the intraclass correlation coefficient. To assess observational studies employing cross-sectional and longitudinal designs, we used the modified Newcastle–Ottawa Scale [47], which included seven items consisting of three quality components: selection (4 items, maximum 5 points), comparability (1 item, maximum 2 points), and outcome (2 items, maximum 3 points). Each criterion received zero to two points and summed to a final score (maximum 10 points). The methodological quality of a specific study was considered high if it was scored as 9 to 10, medium if scored as 5 to 8, and low if scored as 4 or less. To assess studies involving RCTs, we used the Effective Public Health Practice Project (EPHPP) Quality Assessment Tool [18], which included six quality components: selection bias, design, confounders, blinding, data collection, withdrawal and dropout. An overall rating was determined based on the ratings of the above constructs. RCT studies were categorized as high quality if no weak ratings were present, medium if there was only one weak rating, and low if there were two or more weak ratings.

### Data extraction

All data from the included records were extracted by one reviewer (BL) and double checked by a second reviewer (JY). For each study, we coded the following bibliographic information: (1) first author’s name, (2) publication year, and (3) study location. We then coded the following variables from each study: (1) study design type (observational or experimental), (2) participant characteristics (age, sample size, and the number of girls and boys), (3) measures and outcomes of PA (e.g., custom questionnaire, the accelerometer) and MH, (4) main findings (relationship between PA and MH). Follow-up time was extracted for longitudinal studies, and experimental conditions and intervention components, were extracted for experimental studies.

### Evidence synthesis

The relationship between PA and MH in different age ranges was determined by examining the percentage of studies that reported a statistically significant relationship [48]. Referring to previous studies [16, 50] and the definition of age ranges of youth by United Nations [49], participants were categorized as follows: age group 1 (2–5 years), age group 2 (6–12 years), age group 3 (13–18 years), and age group 4 (19–24 years). The relationship between PA and MH in participants with DCC was synthesized separately.

The coding rules were: MH outcomes with different terms but the same concepts were combined into a single identification factor. If a study examined the relationship between PA and one or more sub-dimensions of an MH outcome and most of the sub-dimensions had consistent associations with PA, this could be summarized as a general result of the association of PA with that MH outcome. Various statistical techniques (e.g., t-test, analysis of variance, linear regression, and logistic regression) were used in the included studies to estimate the associations between MH and PA outcomes. For each article, a statistically significant relationship between MH and PA was coded as “relevant” while a statistically insignificant one was coded as “irrelevant” [48]. A summary code of the relationship between each MH outcome and PA was obtained by dividing the number of findings supporting a specific MH outcome associated with PA by the total number of studies that examined the relationship between PA and that particular MH outcome. If 0–33% of the studies reported a statistically significant relationship between PA and MH, the result was categorized as “no association” (0). If 34–59% of the studies reported a statistically significant relationship between PA and MH, the result was categorized as “inconsistent” or “uncertain” (?). If 60–100% of the studies reported a positive or negative relationship between PA and MH, the result was coded as a “positive association” or “negative association” (+)/(–). Double summary codes were indicated as (00), (??), (+ +), or (– –) when three or more studies consistently supported no association, inconsistent, or positive or negative association, respectively. Evidence for the relationship between a specific MH outcome and PA was considered as sufficient only if such relationship obtained a double summary code.

### Meta-analysis

We selected a minimum of ten studies investigating the same MH dimension or outcome for the meta-analysis. All values of the correlations between PA and MH were transformed into Fisher’s z scores and eventually used in the meta-analysis. The Fisher transformation of the correlation coefficient was chosen because the assumption of normality of the results obtained after the transformation would be more plausible [51]. The Fisher’s z transformation was a two-step process that first converted the relevant data describing the relationship between PA and MH (e.g., regression coefficient β, odds ratio, or other effect sizes) into a correlation coefficient r using Psychometrica (https://www.psychometrica.de/effect_size.html). This step was not necessary if the correlation coefficient r was reported in the study. Using the online platform Practical Meta-Analysis Effect Size Calculator, we transformed all correlation coefficient r values to Fisher’s z scores to obtain standardized data for conducting the subsequent meta-analysis (http://www.campbellcollaboration.org/escalc/html/EffectSizeCalculatorHome.php). When an article reported multiple quantitative values of the relationship between PA and MH (e.g., the correlation coefficient between PA and MH for boys and girls was reported separately), we calculated Fisher’s z scores for boys and girls separately based on the reported values and the corresponding number of participants. Fisher’s z scores of 0.12, 0.24, and 0.41 were interpreted as a small, medium, and large effect, respectively [36].

The Stata software version 16.0 (Stata Corp, College Station, TX, USA) was used to perform the meta-analysis. Owing to the anticipated heterogeneity across studies, we conducted a random-effects model. Heterogeneity was quantified with Q statistic and I^2^. Q test (*p* < 0.10) and an I^2^ > 75% indicated a significant high-level heterogeneity [52]. When a high-level heterogeneity appeared, subgroup analyses were performed. If the subgroup analyses could not resolve the high heterogeneity, appropriate univariate meta-regression analyses were performed to explore potential influencing factors, including age of participants, report of total and dimensions (duration, frequency, intensity) of PA, study design (interventional, observational), and disability population group [52, 53]. Additionally, funnel plots and Egger’s tests were performed to assess the risk of publication bias. Funnel plots provided a visual representation of the symmetric distribution of the studies. When the funnel plot was asymmetric, Egger’s tests were conducted to further assess the risk of publication bias. If the Egger’s test was significant (*p* < 0.05), the trim-and-fill method was used to adjust for the suspected publication bias and recalculate the pooled effect size [54, 55].

## Results

### Study selection

**Fig. 1:**
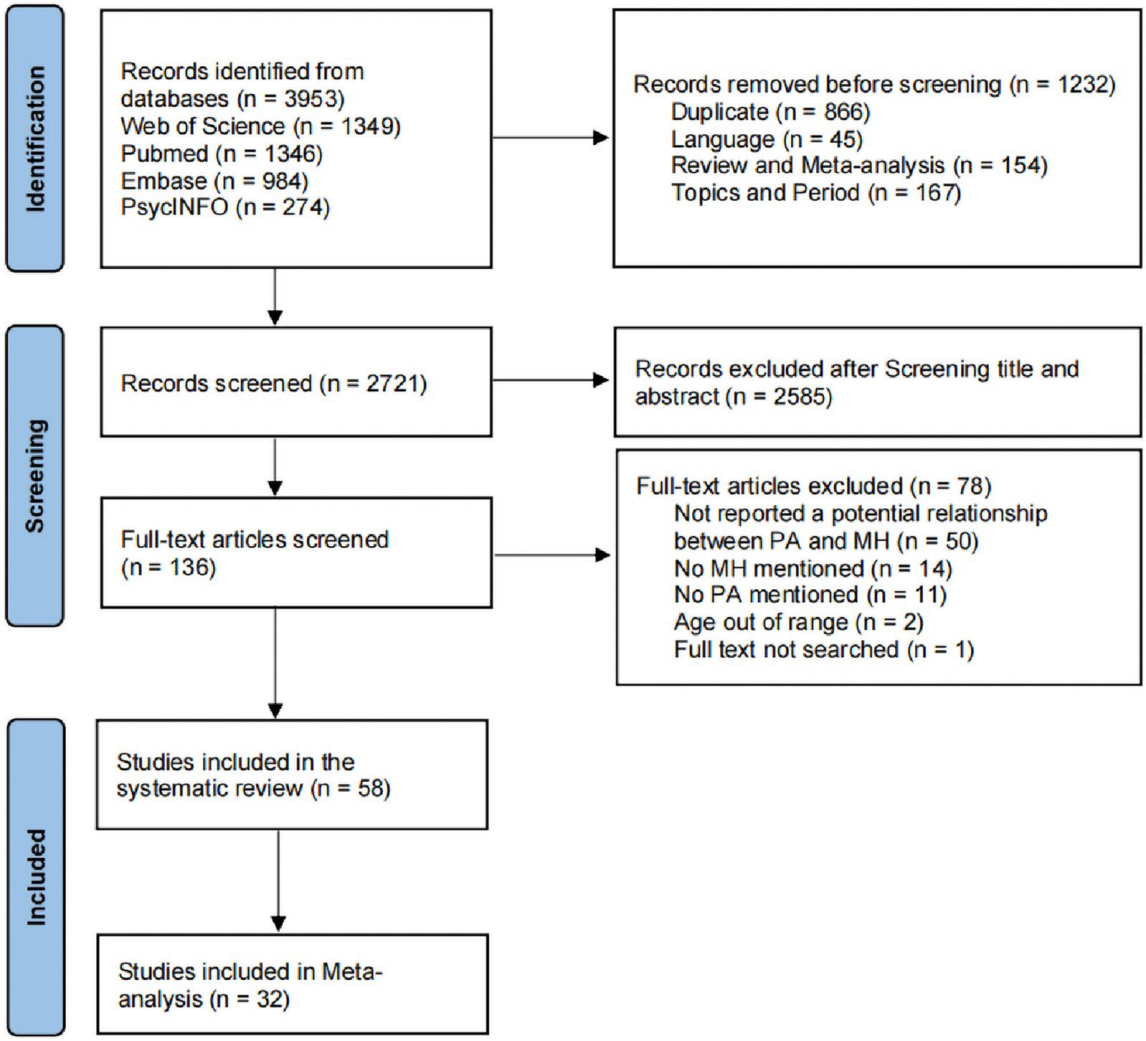
Flow diagram of selection process. *MH* mental health, *PA* physical activity

The search yielded a total of 3953 records. After removing duplications and screening for titles, abstracts, and full-texts, 58 records were included in this systematic review for categorization of variables, with 32 of them applicable for the meta-analysis. The inter-rater reliability between the two reviewers was good for the title and abstract screening (κ = 0.88) and full-text screening (κ = 0.82). The flowchart for the selection process is shown in Fig. 1.

### Overview of studies

This systematic review included 58 articles conducted in 18 countries: China (n = 23), The United States (n = 7), Canada (n = 6), Italy (n = 3), Korea (n = 3), Brazil (n = 2), Germany (n = 2), The United Kingdom (n = 2), Bangladesh (n = 1), The Czech Republic (n = 1), Greece (n = 1), Hungary (n = 1), Iran (n = 1), Israel (n = 1), Lithuania (n = 1), Pakistan (n = 1), Poland (n = 1), and Saudi Arabia (n = 1). Of the included studies, 49 (84%) were published in 2021 (first quarter, n = 6; second quarter, n = 11; third quarter, n = 12; fourth quarter, n = 20), 51 were cross-sectional, four were experimental, and three were longitudinal. The number of participants ranged from 64 to 1,199,320, and their ages ranged from 2 to 24 years old. The most frequently studied age range was 13–18 years (n = 38), followed by 6–12 years (n = 22), 19–24 years (n = 16), and 2–5 years (n = 2). Additionally, 13 of the 58 included studies involved individuals with DCC (e.g., obesity, ADHD, mental illness).

Regarding the measurement of PA, 29 (50%) of the included studies used custom questionnaires, 27 (47%) used known questionnaires (e.g., International Physical Activity Questionnaire) with psychometric properties reported, and 4 (7%) used device based measures. The majority (n = 52, 90%) of included studies used validated questionnaires or scales to evaluate MH outcomes. The top five most frequently used measurements of MH included Patient Health Questionnaire-9 (n = 6), The Depression Anxiety Stress Scale (n = 6), Generalized Anxiety Disorder Scale-7 (n = 5), Center for Epidemiologic Studies Depression Scale (n = 5), and Strengths and Difficulties Questionnaire (n = 4). Additionally, eight included studies either directly selected sub-scale(s) from a validated measurement or developed a new scale based on an existing measurement with adaptations. For example, the COVID-19 Fear Scale was developed in one study as an adaptation of the SARS Fear Scale [59].

A variety of MH outcomes consisting of 36 negative responses and 16 positive responses were investigated in the included studies. Of the negative responses, depression (n = 23), anxiety (n = 21), and stress (n = 11) were investigated most frequently, followed by insomnia (n = 6), COVID-19 stress (n = 3), fatigue (n = 3), mental health problems (n = 3), negative affect (n = 3), anger (n = 2), boredom (n = 2), confusion (n = 2), distress (n = 2), emotional and behavioral problems (n = 2), loneliness (n = 2), tension (n = 2), tiredness (n = 2), aggressiveness (n = 1), being more stressed (n = 1), COVID-19 fear (n = 1), event-specific distress (n = 1), exercise dependence (n = 1), feeling more horrified (n = 1), feeling more apprehensive (n = 1), feeling more helpless (n = 1), feelings of loss of control (n = 1), having greater study pressure (n = 1), hyperactive-impulsive (n = 1), irritability (n = 1), inattention (n = 1), internalizing and functioning problems (n = 1), negative arousal (n = 1), pessimism (n = 1), perceived vulnerability (n = 1), psychosocial and behavioral problems (n = 1), post-traumatic stress disorder (PTSD) (n = 1), and sadness (n = 1). In contrast, general well-being (n = 13) was the most frequently studied positive response, followed by positive affect (n = 3), self-esteem (n = 3), vigor (n = 3), health-related quality of life (n = 2), life satisfaction (n = 2), resilience (n = 2), happiness (n = 1), mental health performance (n = 1), mental health importance (n = 1), optimism (n = 1), positive energy (n = 1), positive outlook (n = 1), prosocial behavior (n = 1), perceived health (n = 1), and relaxation (n = 1). The details of each included observational and experimental study are summarized in, Tables 1, 2, respectively.

**Table 1:** Summary table for the characteristics and methodological quality of observational studies included in the review

Author, year, country, study design	Age (mean age/age range); Sample size (male/female)	Physical activity measurement tool and reporting source (SR/PR)	Mental health outcome and measurement tool	Main findings	Quality of evidence
Alves, et al., USA, Cross-sectional [56]	9–15 years; 64 (24/40)	CQ (SR)	Positive affect, Anxiety, Negative affect; STAIC, Positive and Negative Affect Schedule for Children	MVPA was associated with reduced anxiety levels in children with overweight/obesity (r = − 0.52, *p* < 0.05)	Medium
Alves, et al., USA, Cross-sectional [57]	9–15 years; 65 (25/40)	CQ (SR)	Anxiety; STAIC	Children who engaged in VPA had lower mean anxiety (β = − 2.8, *p* = 0.01); Children who reported more time spent in MVPA also had lower anxiety (β = − 0.2, *p* = 0.01)	Medium
Awais, et al., Pakistan, Cross-sectional [58]	17.9 ± 1.22 years; 225 (107/118)	GLTEQ (SR)	Distress; Kessler-10	A moderate negative correlation of PA (r = − 0.340, *p* < 0.001) was found with psychological distress levels	Medium
Bosselmann, et al., Germany, Cross-sectional [59]	15.83 ± 1.73 years; 122 (53/69)	GSLTPAQ (SR)	Boredom, COVID-19 Fear; FOC-19, Multidimensional State Boredom Scale	COVID-19 Fear significantly correlated with total PA (r = − 0.214, *p* = 0.017), quantity of strenuous PA (r = − 0.325, *p* < 0.01), and boredom (r = − 0.211, *p* < 0.05)	Medium
Breidokiene, et al., Lithuania, Cross-sectional [60]	9.65 ± 1.94 years; 306 (144/162)	CQ (PR)	General well-being; CQ	There was no significant correlation between PA and a Child’s emotional well-being/behavior	Low
Chi, et al., CHN, Cross-sectional [61]	15.26 ± 0.47 years; 1794 (1,077/787)	IPAQ-SF (SR)	Anxiety, Depression, Insomnia; YSIS; PHQ-9; GAD	Moderately active physically was significantly associated with a lower level of depressive symptoms (β = − 0.16, *p* = 0.002) and anxiety symptoms (β = − 0.16, *p* = 0.005), while highly active physically was associated with a lower level of insomnia symptoms (β = − 0.05, *p* = 0.020), depressive symptoms (β = − 0.17, *p* < 0.001) and anxiety symptoms (β = − 0.15, *p* = 0.004)	Medium
Constantini, et al., Israel, Cross-sectional [62]	17.4 ± 0.8 years; 473 (302/171)	CQ (SR)	Resilience; CD-RISC	PA was important factor associated with resilience (β = 0.008, *p* < 0.001)	Medium
Cosma, et al., Czekh, Cross-sectional [63]	13.45 ± 1.62 years; 3438 (1574/1866)	CQ (SR)	General well-being, Life satisfaction; WHO-5, Cantril ladder	Sports and PA components were positively associated with WHO-5 (β = 0.158, *p* < 0.001) and Life Satisfaction (β = 0.110, *p* < 0.001)	Medium
Deng, et al., CHN, Cross-sectional [64]	18–22 years; 1607 (1041/566)	CQ (SR)	Anxiety, Depression, Stress; DASS-21	Lower DASS-21 scores were significantly correlated with regular exercise, maintaining exercise habits during the outbreak of COVID-19, exercising more than 1 to 2 times a week, exercise duration > 1 h, and > 2000 pedometer steps (all *p* < 0.05)	Medium
Ellis, et al., CAN, Cross-sectional [65]	16.68 ± 0.78 years; 1054(231/805) (18 people who chose the “other”)	GLTEQ (SR)	Depression, Loneliness, COVID-19 Stress; CQ, Brief Symptom Inventory, UCLA Loneliness Scale	PA significantly correlated with Loneliness (r = − 0.12, *p* < 0.01) and depression (r = − 0.08, *p* < 0.05). More PA (β = − 0.09, *p* < 0.001) was significant predictor of less loneliness	Medium
Fang, et al., CHN, Cross-sectional [66]	20.17 ± 0.47 years; 1011 (328/683)	The five-item scale was developed by Boothby, Tungatt, and Townsend (SR)	General well-being; The eight-item scale developed by Campbell, The 28-item scale developed by Burckhardt and Anderson	Participation in sports was positively related to general well-being (r = 0.02, *p* < 0.01). The linear regression analysis of how participation in sports influences well-being (β = 0.25, *p* < 0.001) revealed a significant amount of explained variance	Medium
Ghorbani, et al., Iran, Cross-sectional [67]	16.28 ± 0.97 years; 136 (60/76)	The accelerometer ActiGraph wGT3X-BT	Anxiety, Depression, Stress; DASS-21	The analyses showed that MVPA per day was negatively associated with all of the mental health indicators; depression (β = − 0.290, *p* = 0.001), anxiety (β = − 0.404, *p* < 0.001), and stress (β = − 0.298, *p* < 0.001)	Medium
Gilbert, et al., USA, Cross-sectional [68]	8.01 ± 1.75 years; 144 (80/64)	CQ (PR)	General well-being; Child Mood and Feelings Questionnaire,	Children with a decrease in total PA, outside play and organized sports programs had lower general well-being (*p* < 0.05)	Medium
Huang, et al., CHN, Cross-sectional [69]	NR, Middle school students; 1493 (778/715)	CQ (SR)	Negative affect, Feelings of loss of control; CQ	There were significant differences among students with low, medium, and high PA in terms of their self-perceived feelings of loss of control (*p* < 0.01, effect size = 0.274) and negative affect (*p* < 0.01, effect size = 0.257)	Medium
Kang, et al., CHN, Cross-sectional [70]	16.3 ± 1.3 years; 4898 (2359/2539)	IPAQ (SR)	Vigor, Self-esteem, Tension, Depression, Anger, Fatigue, Confusion; Chinese Profile of Mood States	Higher levels of PA were significantly associated with lower levels of total mood disturbance in this population (High PA group according to IPAQ: B = − 3.22, SE = 0.40, *p* < 0.001; Moderate: B = − 1.47, SE = 0.37, *p* < 0.001, compared to Low PA group)	Medium
Khan, et al., Bengladesh, Cross-sectional [71]	NR, college and university students; 505 (317/188)	CQ (SR)	Anxiety, Depression, Stress, Event-specific distress; DASS-21, Impact of Event Scale	Physical exercise was significantly associated in lowering scores of DASS depression subscale (B = − 2.10, *p* < 0.05)	Medium
Lai, et al., CHN, Cross-sectional [72]	NR, University Students; 124 (45/79)	CQ (SR)	Resilience, Anxiety, Depression, insomnia; Stress; PSS-10, PHQ-4, The seven-item Insomnia Severity Scale, CD-RISC	Exercise was significantly related to anxiety and depression (r = − 0.194, *p* < 0.05)	Medium
Lee, Korea, Cross-sectional [73]	14–19 years; 1,046 (521/525)	Model of sports participation developed by Snyder	COVID-19 Stress; COVID-19 Stress Scale	PA was significantly related to COVID-19 stress (r = − 0.162, *p* < 0.001)	Medium
Lee, et al., Korea, Cross-sectional [74]	14–16 years; 333 (153/180)	Ware’s “Health Perception” Scales (SR)	Mental health importance, Mental health performance; Ware’s “Health Perception” Scales	PA performance was significantly related to mental health importance (r = 0.533, *p* < 0.001), mental health performance (r = 0.520, *p* < 0.001)	Medium
Li, et al., CHN, Cross-sectional [75]	5.21 ± 1.40 years; 21,526 (11,281/10,245)	CQ (PR)	Mental health problems; SDQ	PA < 1 h/day (OR: 1.21, *p* < 0.001) was associated with increased risks for child mental health problems	Medium
Lindoso, et al., Brazil, Cross-sectional [76]	10–18 years; 355 (140, 215)	CQ (SR)	Mental health problems; SDQ	There was a negative correlation between the mental health problems score and PA per week (r = -0.222, *p* < 0.001)	Medium
Lu, et al., CHN, Cross-sectional [77]	15.26 ± 0.46 years; 965 (556, 409)	IPAQ-SF (SR)	Anxiety, Depression, COVID-19 Fear, Insomnia; YSIS, PHQ-9, GAD, FOC-19	People with high PA were less likely to experience insomnia (OR = 0.71, *p* < 0.05) and depression (OR = 0.71, *p* < 0.05) compared to those with low PA	Medium
Lukacs, Hungary, Cross-sectional [78]	24.52 ± 7.15 years; 2162 (599/1552) (11 people who chose the “other”)	CQ (SR)	General well-being, Perceived health; CES-D	The test indicated students with reduced MVPA had lower general well-being and perceived health status scores than students who were unchanged (*p* < 0.001) or increased their activities (*p* < 0.001)	Medium
Masse, et al., CAN, Cross-sectional [79]	13 ± 0.1 years; 254 (117/ 137)	CQ (SR)	General well-being, Anxiety; CQ	Children's general well-being had a significant impact on PA (β = 0.286, 95% CI 0.138–0.424, *p* < 0.05)	Medium
Maximova, et al., CAN, Cross-sectional [80]	9-12 years; 1095 (538/557)	CQ (SR)	General well-being, Positive outlook, Internalizing and functioning problems, Tiredness, Loneliness, Boredom; CQ	Girls who were more physically active during than before the lockdown were less likely to experience ‘internalizing and functioning problems, tiredness and loneliness’ and more likely to have a ‘positive outlook on future and time during lockdown’ relative to those who were less physically active. Boys who were physically active during the lockdown were more likely to have a ‘positive outlook on future and time during the lockdown’	Medium
McArthur, et al., CAN, Cross-sectional [81]	9–11 years; 846 (447/398)	CQ (SR)	Happiness, Anxiety, Depression; CQ	PA was significantly related to anxiety (r = − 0.11, *p* < 0.05) and happiness (r = 0.15, *p* < 0.05)	Medium
McGuine, et al., USA, Cross-sectional [82]	15.7 ± 1.2 years; 559 (313/244)	The Hospital for Special Surgery Pediatric Functional Activity Brief Scale (SR)	Health-related quality of life, Anxiety, Depression; GAD-7, PHQ-9, Pediatric Quality of Life Inventory 4.0	The did not play (DNP) group had a higher (ie., worse) GAD-7 score than the did play (PLY) group (*p* < 0.001) as well as a higher (ie., worse) PHQ-9 score than the PLY group (*p* < 0.001). the PLY group had a higher Pediatric Quality of Life Inventory total scores (*p* < 0.001)	Medium
Mitra, et al., CAN, Cross-sectional [83]	9–15 years; 800 (377/423)	CQ (SR)	General well-being ; CQ	Lower general well-being was related to less PA (OR = 1.54, *p* = 0.028)	Medium
Moriarty, et al., USA, Cross-sectional [84]	21.3 ± 3.8 years; 550 (135/408)	IPAQ-SF (SR)	Stress; PSS-4	Stress was negatively associated with exercise during COVID-19 (r = − 0.162, *p* < 0.001)	Medium
Morres, et al., Greece, Cross-sectional [85]	14.41 ± 1.63 years; 950 (518/432)	IPAQ-SF (SR)	Positive energy, Relaxation, General well-being, Negative arousal, Tiredness; The 4-Dimensional Mood Scale, WHO-5	Total PA was significantly related to general well-being (r = 0.35, *p* < 0.01), positive energy (r = 0.36, *p* < 0.01), relaxation (r = 0.12, *p* < 0.01), and negative arousal (r = − 0.10, *p* < 0.01)	Medium
Oliva, et al., Italy, Cross-sectional [86]	1-18 years; 9688 (5066/4622)	CQ (PR)	Anxiety, Depression, Emotional and behavioral problems; The Pediatric Symptom Checklist, CES-D, The Screen for Child Anxiety Related Disorders	Emotional and behavioral problems (Estimate = − 5.7980, *p* < 0.001), anxiety (Estimate = − 4.3827, *p* < 0.001), and depression (Estimate = − 3.0091, *p* < 0.001) were negatively correlated with PA	Medium
Pigaiani, et al., Italy, Cross-sectional [87]	18.1 ± 0.9 years; 306 (223/83)	CQ (SR)	General well-being; CQ	PA was significantly related to general well-being (OR = 2.609, *p* = 0.007)	Medium
Qi, et al., CHN, Cross-sectional [88]	11–20 years; 9554 (4557/4997)	CQ (SR)	Depression; CES-D	Duration of PA > 60 min/day (OR = 0.686, *p* < 0.001) and 30–60 min/day (OR = 0.636, *p* < 0.001) were significantly associated with lower risk of depression	Medium
Qin, et al., CHN, Cross-sectional [89]	13–16 years; 248 (114/134)	CQ (SR)	Anxiety; Mental Health Test	Exercising for ≥ 1 h per day (OR = 0.23, *p* < 0.01) was a protective factor for anxiety	Medium
Qin, et al., CHN, Cross-sectional [90]	12.04 ± 3.01 years; 1,199,320 (619,144/580,176)	CQ (SR)	Distress; GHQ-12	Students who spent less than 0.5 h exercising had increased odds of self-reported psychological distress compared with students who spent more than 1 h exercising (OR = 1.64, *p* < 0.001)	High
Ren, et al., CHN, Cross-sectional [91]	13.14 ± 1.55 years; 1487 (727/760)	CQ (SR)	Depression; CES-D	PA time (β = − 0.07, *p* < 0.001) was negatively associated with depressive symptoms	Medium
Sikorska, et al., Poland, Cross-sectional [92]	15.38 ± 2.10 years; 455 (121/243) (2 non-binary people and 4 people who chose the “other”)	CQ (SR)	Resilience, General well-being, Anxiety, Depression, Stress; CD-RISC, DASS	Physical exercise was significantly associated resilience (r = 0.183, *p* < 0.01), emotional well-being (r = 0.153, *p* < 0.01), psychological well-being (r = 0.191, *p* < 0.01), Social well-being (r = 0.126, *p* < 0.05)	Medium
Song, et al., Korea, Cross-sectional [93]	NR, middle school and high school students; 836 (412/424)	Social Aspect of Sport (SR)	Optimism, Pessimism, COVID-19 Stress; A scale with Verified Reliability and Validity as Reported by Chang, The scales Used by Gaumer Erickson, The COVID Stress Scale	Sports participation exerted a positive effect on optimism (β = 0.659, *p* < 0.001). Sports participation exerted a negative effect on pessimism (β = − 0.156, *p* = 0.037) and COVID Stress (β = − 0.656, *p* < 0.001)	Medium
Swansburg, et al., CAN, Cross-sectional [94]	10.14 ± 3.06 years; 587 (166/412) (9 people who chose the “other”)	A daily activities table with the same activity categories as the United Kingdom Co-SPACE study (SR)	Anxiety, Depression, Hyperactive impulsive, Inattention; PHQ-9, GAD, The Swanson, Nolan, and Pelham 26-question scale	Exercising < 1 h/day correlated positively with the PHQ-9 (r = 0.110, *p* < 0.01) but negatively with the hyperactive/impulsive (r = − 0.086, *p* < 0.05) score	Medium
Szwarcwald, et al., Brazil, Cross-sectional [95]	12-17 years; 9470 (4716/4754)	The National School Health Survey (SR)	Insomnia, Irritability, Sadness; World Health Survey	PA for 60 min or over at least twice a week was inversely correlated with the problem (At least two problems from frequent sadness, frequent irritability, and sleep problems) (OR = 0.82, *p* < 0.001)	Medium
Tandon, et al., USA, Cross-sectional [96]	10.8 ± 3.5 years; 1000 (517/467)	CQ (SR)	Mental health problems; SDQ	For younger children (6–10 years), engaging in the recommended 7 day/week of PA was associated with mental health problems (β = − 2.4, *p* = 0.04). For older children (11–17 years), engaging in 1–6 (β = − 3.5, *p* < 0.01), and 7 day/week (β = − 3.6, *p* < 0.01) of PA was significantly associated with mental health problems	Medium
Thomas, et al., United Kingdom, Longitudinal [97]	16–24 years; 64 (34/30)	CQ (SR)	Self-esteem, General well-being; The Short Warwick-Edinburgh Mental Wellbeing Scale	Changes in general well-being were positively associated with changes in moderate PA (r = 0.24, *p* < 0.05), and total PA (r = 0.28, *p* < 0.05)	Medium
Wang, et al., CHN, Cross-sectional [98]	NA, primary scholar; 6017 (3287/2730)	CQ (PR)	Emotional and behavioral problems; SDQ	Exercise duration of 30 min/day could reduce the risk of Emotional and behavioral problems	Medium
Wang, et al., CHN, Cross-sectional [99]	6–16 years; 12,186 (6357/5829)	CQ (SR)	Psychosocial and behavioral problems; The Child Behavior Checklist Score	PA time per day was a significant risk factor associated with mood and behavior problems: outside Wuhan (OR = 0.510, *p* < 0.001), within Wuhan (OR = 0.416, *p* < 0.01)	Medium
Wang, et al., USA, Longitudinal [100]	13–18 years; 349 (140/209)	GLTEQ (SR)	Positive Affect, Stress; The Multicultural Events Schedule for Adolescents, The Positive and Negative Affect Scale for Children	Daily exercise was significantly associated Stress (r = − 0.05, *p* < 0.001) and positive affect (r = 0.23, *p* < 0.001)	Low
Wright, et al., United Kingdom, Cross-sectional [101]	15.9 ± 1.48 years; 165 (65/100)	CQ (SR)	Vigor, Anxiety, Depression, Fatigue, Stress; PSS-10, The Hospital Anxiety and Depression Scale, The 20-item Multidimensional Fatigue Inventory, The Subjective Vitality Scale	Higher levels of PA were associated with lower levels of stress (r = -0.26, *p* < 0.01), depression (r = − 0.31, *p* < 0.01), fatigue (r = − 0.38, *p* < 0.01), as well as higher levels of vigor (r = 0.34, *p* < 0.01)	Medium
Wunsch, et al., Germany, Cross-sectional [102]	10.36 ± 4.04 years; 1711 (750/961)	The Momo Physical Activity Questionnaire (SR)	Health-related quality of life, The KIDSCREEN-10 Index	PA within-COVID-19 was positively predicted by pre-COVID-19 health-related quality of life (standardized estimate = 0.07; *p* = 0.003)	Medium
Xu, et al., CHN, Cross-sectional [103]	NA, college and university students; 11,254 (4054/7200)	CQ (SR)	Anxiety, Depression, Insomnia, Posttraumatic stress disorder (PTSD); PHQ-9, GAD-7, ISI, Posttraumatic Stress Disorder Checklist for DSM-5	Participants who engaged in regular exercise during the pandemic reported a lower risk of symptoms of depression (OR = 0.86, *p* = 0.01), and insomnia (OR = 0.82, *p* < 0.01)	Medium
Zhang, et al., CHN, Cross-sectional [104]	NA, college and university students; 2270 (877/1393)	CQ (SR)	Anxiety, Depression; The Self-Rating Anxiety Scale, The Self-Rating Depression Scale	Exercise during the epidemic outbreak (OR = 0.456, *p* < 0.001) was protective factors for anxiety and depression	Medium
Zhang, et al., CHN, Cross-sectional [105]	11.63 ± 1.23 years; 9979 (5131/4848)	IPAQ-SF (SR)	Self-esteem, Vigor, Anger, Confusion, Depression, Fatigue, Tension; Chinese Profile of Mood States	Moderate PA (β = − 3.031,* p* < 0.001) and High PA (β = − 1.309, *p* = 0.047) were significantly correlated with mood states in children and adolescents	Medium
Zhang, et al., CHN, Cross-sectional [106]	20.51 ± 1.88 years; 11,787 (5056/6731)	CQ (SR)	Depression; PHQ-9	Compared with PA ≥ 3 day/week, PA < 3 day/week was positively associated with depression symptoms (β = 0.01, 95% CI 0.008–0.012)	Medium
Zhang, et al., CHN, Longitudinal [107]	20.70 ± 2.11 years; 66 (25/41)	IPAQ (SR)	Aggressiveness, Anxiety, Depression, Stress; DASS-21, The Buss-Perry Aggressive Questionnaire	Each 100-unit increase in METs of total PA corresponded to a change of (Point Estimate = − 0.12, *p* < 0.05) in the global DASS score. PA also significantly alleviated depression (Point Estimate = − 0.04, *p* < 0.05)	Medium
Zhou, et al., CHN, Cross-sectional [108]	11–18 years; 4805 (0/4805)	CQ (SR)	Depression; CES-D	Compared with physical exercise duration/day of > 30 min, duration/day of < 30 min (OR = 1.641, *p* < 0.001) was a risk factor for depression	Medium
Zhu, et al., CHN, Cross-sectional [109]	12.6 ± 1.3 years; 2860 (1346/1502) (12 people missing)	The Mental Health Lifestyle Scale (SR)	Being more stressed, Feeling more horrified, Feeling more apprehensive, Feeling more helpless, Having larger study pressure, Perceived vulnerability; CQ, The adapted Perceived Risk of the HIV Scale	Those who perceived more vulnerability were more likely than others to spend more time to exercise (OR = 1.28, *p* < 0.001)	Medium

*β* regression coefficient, *CAN* Canada, *CD-RISC* The Connor-Davidson resilience scale, *CQ* Custom questionnaire (questionnaire or survey questions designed by the researcher), *CES-D* The Center for Epidemiological Studies Depression Scale, *CHN* China, *CI* confidence interval,* DASS* Depression, Anxiety, Stress Scale-21, *FOC-19* Fear of COVID-19 Scale, *GAD* The Generalized Anxiety Disorder scale, *GHQ-12* 12-item General Health Questionnaire, *GLTEQ* Godin Leisure-Time Exercise Questionnaire, *GSLTPAQ* Godin-Shephard Leisure-Time Physical Activity Questionnaire, *h* hour, *IPAQ-SF* International Physical Activity Questionnaire Short Form, *ISI* Insomnia Severity Index, *LPA* Light-intensity physical activity, *MVPA* Medium to vigorous physical activity, *OR* The odds-ratio, *PA* physical activity, *PHQ-9* The 9-item Patient Health Questionnaire, *PR* Parents-reported, *PSS-10*, The ten-item Perceived Stress Scale-10, *r* correlation coefficient, *SDQ* Strengths and Difficulties Questionnaire, *SR* Self-rated; *STAIC* State-Trait Anxiety Inventory for Children, *t* t-statistic, *USA* United States, *VPA* Vigorous-intensity physical activity, *WHO-5* The World Health Organization Well-Being Index-5, *YSIS* Youth Self-Rating Insomnia Scales*COVID‐19* the coronavirus disease, *PA* physical activity, *MH* mental health, *RCTs* randomized controlled trials, *DCC* disabilities or chronic conditions

**Table 2:** Summary table for the characteristics and methodological quality of experimental studies included in the review

Country, study design	Age (mean age/age range); Sample size (male/female), Participants Characteristics (types of disorders or diseases), Grouping	Condition for the control, Condition for the intervention, Intensity, frequency, duration	Mental health outcome and measurement tool	Main findings	Quality of evidence
Chen, et al., CHN, Randomized controlled trials [110]	14.4 ± 1.0 years; 69, patients with moderate and severe anxiety symptoms (anxiety Scale ≥ 61), experiment group (35) and the control group (34)	The control group was given routine health education support the experiment group was given both routine health education support and the integration model for intervention (mindfulness meditation training, aerobics exercise course). 30 min (mindfulness meditation training); 45 min, medium intensity (aerobics exercise course); 5 times a week (mindfulness meditation training); 3 times a week (aerobics exercise course); 8 weeks (mindfulness meditation training); 10 weeks (aerobics exercise course)	Positive Affect, General well-being, Life satisfaction, Anxiety, Negative Affect; Self-rating Anxiety Scale, Positive and Negative Affect Scale, Psychological Well-Being Scale	After intervention, a significant difference between groups was obtained in anxiety scores, negative affect scores, positive affect scores (all *p* < 0.01), and overall well-being index (*p* = 0.04)	Medium
De Candia, et al., Italy, Randomized controlled trials [111]	16.13 ± 0.74 years; 50, NA, information regarding nutritional education (EG; n = 25) or a waitlist control group (CG; n = 25)	Do nothing. Receive 12 weeks of aerobic exercises characterized by fun elements associated with theoretical lessons 90 min (physical activities, such as joint mobility exercises, low-to-moderate intensity aerobic exercise, team-building activities, exercise stations, cardio workout) 2 times a week; 12 weeks	Exercise Dependence, Stress; Exercise Dependence Scale, PSS-10	The post-hoc analysis revealed a significant improvement in the score for exercise dependence (*p* < 0.001, d = 2.05, large effect size) and stress (*p* < 0.001, d = 2.17, large effect size) in the intervention group	Medium
Hamed, et al., Saudi Arabia, Randomized controlled trials [112]	20.77 ± 1.16 years; 54, mild to moderate anxiety and depression, participants were divided into: group A (GA) and Group B (GB)	Group B (GB) students received an online CBT (1.5 h each session) for 8 weeks (once per week). Group A (GA) were encouraged to increase their aerobic training of moderate to vigorous exercises such as jumping, running, swimming, or dancing, 5 days/week for one h daily.80 min, 70–90% of their maximum heart rate; 5 times a week; 8 weeks (tread mill running, high pace stationary cycling or weight bearing aerobic exercises)	Anxiety, Depression, Stress; DASS-21	A significant improvement of DASS scores after treatment in both groups (*p* ≤ 0.001). IPAQ scores showed a significant improvement in GA and GB with non-significance in vigorous activities; category. GA showed a significant reduction of anxiety more than GB with a non-significant difference in stress and depression (*p* ≥ 0.05)	High
Zheng, et al., CHN, Randomized controlled trials [113]	13.5 ± 0.5 years; 954(499/455), NA, intervention (n = 485, 6 schools) and control (n = 469, 6 schools) groups	(1) An outline was provided on the recommended 20-20-20 rule during study and viewing of on-screen content; (2) During recess (15 min for each recess; 4 times per day), participants in the control group received SMS text message prompts (≤ 50 characters) to participate in broadcast exercise programs at home, eye relaxation, or to stretch for 10 min. Students had access to at-home workout videos developed by exercise physiologists. Students in the intervention group received the identical health information session, online curriculum, workout videos, and breaks as described above. Additionally, at the beginning of the study, students in the intervention group were asked to log on and download a peer-to-peer live-streaming app (the Recess and Exercise Advocacy Program [REAP]). REAP is a live-streaming platform that allows users to capture short videos and photographs with their smartphones related to their physical exercise or eye relaxation activities (eg., looking outdoors through the window).15 mins for each recess, 4 times per day; 2 weeks	Anxiety, Insomnia; The 45-item Chinese version of the Spence Children’s Anxiety Scale, The 4-item Patient-Reported Outcomes Measurement Information System	Anxiety score fell by − 0.23 in the intervention group and rose (worsened) by 0.12 in the controls by the end of the study. The change in anxiety score was significantly greater in the intervention group compared to the controls (− 0.36, *p* = 0.02)	High

*CHN* China, *d* Cohens d, *DASS-21* Depression, Anxiety, Stress Scale-21, *h* hour,*IPAQ* International Physical Activity Questionnaire,*PA* physical activity, *PSS-10* The ten-item Perceived Stress Scale-10

### Quality assessment

Of the 58 included studies, 53 (91%) were rated as medium in the quality assessment, three were rated as high, and two were rated as low. For the methodological quality ratings (see Tables 1, 2), the inter-rater reliability between the two reviewers was good (intraclass correlation coefficient = 0.77).

### Data syntheses

#### Changes in physical activity and mental health of children and youth during COVID-19

In the present review, 11 included studies reported a significant decrease in PA among children and youth during COVID-19. For example, one study revealed that only 3.6% of children (5–11 years) and 2.6% of youth (12–17 years) in Canada participated in moderate to vigorous PA for at least 60 min per day during COVID-19 [114]. Another study showed that Spanish children spent 91 min per day less on PA during COVID-19 than before [25]. Such PA reductions were reflected in various components, including duration, frequency, and intensity [57, 68, 78, 80, 83, 84, 115–117].

Regarding the impact of COVID-19 on MH, 25 included studies showed that the MH of children and youth deteriorated during the pandemic. For example, one study found that COVID-19 caused significantly elevated levels of anxiety in youth [118], while another showed an increase in depressive symptoms in the 6–12- and 13–18-year-old groups [91]. Additional studies reported a significant increase in psychological distress [119] and an indirect increase in stress levels [107] among youth. As mentioned earlier, COVID-19 not only impacted common negative responses such as depression, anxiety, and stress among children and youth, but also led to increased occurrences of less common mental problems. One study, for instance, showed that during COVID-19, children and youth became more attached, inattentive, and irritable, and preschoolers were more likely to manifest signs of clinginess and fear [120]. Positive responses were also affected, including significant decreases in levels of well-being and overall mental health among children and youth [68, 78, 80, 121]. It is worth noting that children and youth with DCC exhibited a higher incidence of severe psychosocial dysfunction and a lower level of PA compared to their peers without DCC [56, 57, 76, 88, 108].

#### The relationship between physical activity and mental health among children and youth during COVID-19

##### Overall findings

As shown in Table 3, PA was strongly and negatively correlated with depression (21 of 23 studies, 91.3%), anxiety (14 of 21 studies, 66.7%), stress (7 of 11 studies, 63.6%), insomnia (4 of 6 studies, 66.7%), fatigue (3 of 3 studies, 100%), and mental health problems (3 of 3 studies, 100%). Although PA was negatively related to COVID-19 stress, negative affect, anger, confusion, distress, emotional and behavioral problems, COVID-19 fear, exercise dependence, feelings of loss of control, irritability, negative arousal, pessimism, psychosocial and behavioral problems, and sadness, and positively related to hyperactive/impulsive and perceived vulnerability, the evidence was considered insufficient since less than three studies consistently supported a specific association. PA’s relationship with each of the remaining negative response outcomes was either inconsistent or no association. Regarding positive psychological responses, PA showed strong and positive associations with general well-being (11 of 13 studies, 84.6%) and vigor (3 of 3 studies, 100%). Although PA was positively related to positive affect, self-esteem, health-related quality of life, life satisfaction, resilience, happiness, mental health performance, mental health importance, optimism, positive energy, positive outlook, prosocial behavior, perceived health, and relaxation, the evidence was considered insufficient.

**Table 3:** Summary of the relationships between PA and MH outcomes

	Overall				6–12 years group	13–18 years group	19–24 years group	Disability
	Positive relationship	Negative relationship	No relationship	Assoc (% studies)				
Negative psychological responses								
Depression		[61]^b^, [64]^bc^, [65]^b^, [67]^b^, [70]^b^, [71]^c^, [72]^bc^, [77]^b^, [82]^bc^, [86]^abd^, [88]^bcd^, [91]^ab^, [94]^abd^, [101]^bc^, [103]^bc^, [104]^cd^, [105]^ab^, [106]^c^, [107]^c^, [108]^abd^, [121]^bcd^	[81]^a^, [92]^ab^	− – 21/23 (91.3%)	− – 5/7 (83.3%)	− – 17/18 (94.4%)	− – 11/11 (100%)	− – 6/6 (100%)
Anxiety		[56]^abd^, [57]^abd^, [61]^b^, [64]^bc^, [67]^b^, [72]^bc^, [81]^a^, [82]^bc^, [86]^abd^, [89]^b^, [104]^cd^, [110]^bd^, [112]^bcd^, [113]^b^	[71]^c^, [77]^b^, [92]^ab^, [94]^abd^, [101]^bc^, [103]^bc^, [107]^c^,	− – 14/21 (66.7%)	− – 4/6 (66.7%)	− – 12/17 (70.6%)	?? 5/9 (55.6%)	− – 6/7 (85.7%)
Stress		[64]^bc^, [67]^b^, [84]^c^, [98]^b^, [101]^bc^, [111]^b^, [112]^bcd^	[71]^c^, [72]^bc^, [92]^ab^, [107]^c^	− – 7/11 (63.6%)	0 0/1 (0%)	− – 6/8 (75%)	?? 4/7 (57.1%)	− 1/1 (100%)
Insomnia		[61]^b^, [77]^b^, [95]^ab^, [103]^bc^	[72]^bc^, [113]^b^	− – 4/6 (66.7%)	− 1/1 (100%)	− – 4/6 (66.7%)	? 1/2 (50%)	
COVID-19 Stress		[73]^bc^, [93]^b^	[65]^b^	− 2/3 (66.7%)		− 2/3 (66.7%)	− 1/1 (100%)	
Fatigue		[70]^b^, [101]^bc^, [105]^ab^		− – 3/3 (100%)	− 1/1 (100%)	− – 3/3 (100%)	− 1/1 (100%)	
Mental health problems		[75]^a^, [76]^abd^, [96]^ab^		− – 3/3 (100%)	− – 3/3 (100%)	− 2/2 (100%)		− 1/1 (100%)
Negative affect		[69]^b^, [110]^bd^,	[56]^ab^	− 2/3 (66.7%)	0 0/1 (0%)	− 2/3 (66.7%)		− 1/1 (100%)
Anger		[70]^b^, [105]^ab^		− 2/2 (100%)	− 1/1 (100%)	− 2/2 (100%)		
Boredom		[59]^bc^	[80]^a^	? 1/2 (50%)	0 0/1 (0%)	− 1/1 (100%)	− 1/1 (100%)	
Confusion		[70]^b^, [105]^ab^		− 2/2 (100%)	− 1/1 (100%)	− 2/2 (100%)		
Distress		[58]^bc^, [90]^bc^		− 2/2 (100%)		− 2/2 (100%)	− 2/2 (100%)	
Emotional and behavioral problems		[86]^abd^, [98]^ad^		− 2/2 (100%)	− 2/2 (100%)	− 1/1 (100%)		− 2/2 (100%)
Loneliness		[65]^b^	[80]^a^	? 1/2 (50%)	0 0/1 (0%)	− 1/1 (100%)		
Tension			[70]^b^, [105]^ab^	0 0/2 (0%)	0 0/1 (0%)	0 0/2 (0%)		
Tiredness			[80]^a^, [85]^ab^,	0 0/2 (0%)	0 0/2 (0%)	0 0/1 (0%)		
Aggressiveness			[107]^c^	0 0/1 (0%)			0 0/1 (0%)	
Being more stressed			[109]^ab^	0 0/1 (0%)	0 0/1 (0%)	0 0/1 (0%)		
COVID-19 Fear		[59]^bc^		− 1/1 (100%)		− 1/1 (100%)	− 1/1 (100%)	
Event-specific distress			[71]^c^	0 0/1 (0%)			0 0/1 (0%)	
Exercise dependence		[111]^b^		− 1/1 (100%)		− 1/1 (100%)		
Feeling more horrified			[109]^ab^	0 0/1 (0%)	0 0/1 (0%)	0 0/1 (0%)		
Feeling more apprehensive			[109]^ab^	0 0/1 (0%)	0 0/1 (0%)	0 0/1 (0%)		
Feeling more helpless			[109]^ab^	0 0/1 (0%)	0 0/1 (0%)	0 0/1 (0%)		
Feelings of loss of control		[69]^b^		− 1/1 (100%)		− 1/1 (100%)		
Having larger study pressure			[109]^ab^	0 0/1 (0%)	0 0/1 (0%)	0 0/1 (0%)		
Hyperactive/impulsive	[94]^abd^			+ 1/1 (100%)	+ 1/1 (100%)	+ 1/1 (100%)		+ 1/1 (100%)
Irritability		[95]^ab^		− 1/1 (100%)	− 1/1 (100%)	− 1/1 (100%)		
Inattention			[94]^abd^	0 0/1 (0%)	0 0/1 (0%)	0 0/1 (0%)		0 0/1 (0%)
Internalizing and functioning problems			[80]^a^	0 0/1 (0%)	0 0/1 (0%)			
Negative arousal		[85]^ab^		− 1/1 (100%)	− 1/1 (100%)	− 1/1 (100%)		
Pessimism		[93]^b^		− 1/1 (100%)		− 1/1 (100%)		
Perceived vulnerability	[109]^ab^			+ 1/1 (100%)	+ 1/1 (100%)	+ 1/1 (100%)		
Psychosocial and behavioral problems		[99]^ab^		− 1/1 (100%)	− 1/1 (100%)	− 1/1 (100%)		
PTSD			[103]^bc^	0 0/1 (0%)		0 0/1 (0%)	0 0/1 (0%)	
Sadness		[95]^ab^		− 1/1 (100%)	− 1/1 (100%)	− 1/1 (100%)		
Positive psychological responses								
General well-being	[63]^ab^, [66]^bc^, [68]^a^, [78]^c^, [79]^b^, [83]^ab^, [85]^ab^, [87]^bc^, [92]^ab^, [97]^bc^, [110]^bd^		[60]^ab^, [80]^a^	+ + 11/13 (84.6%)	+ + 5/7 (71.4%)	+ + 9/10 (90%)	+ + 4/4 (100%)	+ 1/1 (100%)
Positive affect	[100]^b^, [110]^bd^		[56]^ab^	+ 2/3 (66.7%)	0 0/1 (0%)	+ 2/3 (66.7%)		+ 1/1 (100%)
Self-esteem	[70]^b^, [105]^ab^		[97]^bc^	+ 2/3 (66.7%)	+ 1/1 (100%)	+ 2/3 (66.7%)	0 0/1 (0%)	
Vigor	[70]^b^, [101]^bc^, [105]^ab^			+ + 3/3 (100%)	+ 1/1 (100%)	+ + 3/3 (100%)	+ 1/1 (100%)	
Health-related quality of life	[82]^bc^, [102]^ab^			+ 2/2 (100%)	+ 1/1 (100%)	+ 2/2 (100%)	+ 1/1 (100%)	
Life satisfaction	[63]^ab^, [110]^bd^			+ 2/2 (100%)	+ 1/1 (100%)	+ 2/2 (100%)		+ 1/1 (100%)
Resilience	[62]^b^, [92]^ab^			+ 2/2 (100%)	+ 1/1 (100%)	+ 2/2 (100%)		
Happiness	[81]^a^			+ 1/1 (100%)	+ 1/1 (100%)			
Mental health performance	[74]^b^			+ 1/1 (100%)		+ 1/1 (100%)		
Mental health importance	[73]^b^			+ 1/1 (100%)		+ 1/1 (100%)		
Optimism	[93]^b^			+ 1/1 (100%)		+ 1/1 (100%)		
Positive energy	[85]^ab^			+ 1/1 (100%)	+ 1/1 (100%)	+ 1/1 (100%)		
Positive outlook	[80]^a^			+ 1/1 (100%)	+ 1/1 (100%)			
Prosocial behavior	[98]^ad^			+ 1/1 (100%)	+ 1/1 (100%)			+ 1/1 (100%)
Perceived health	[78]^c^			+ 1/1 (100%)			+ 1/1 (100%)	
Relaxation	[85]^ab^			+ 1/1 (100%)	+ 1/1 (100%)	+ 1/1 (100%)		

In the association column, the strength of evidence was summarized and classified according to the percentage of studies supporting an association: 0–33% coded as “0” indicating no association, 34–59% coded as “?” indicating inconsistent association, 60–100% coded as “ + ” or “ − ” indicating positive or negative association. “00”, “??”, “ +  + ”, “– –” were coded when there were three or more studies supporting an association

*MH* mental health, *PA* physical activity, *PTSD* post-traumatic stress disorder

^a^Includes 6–12 years group

^b^Includes 13–18 years group

^c^Includes 19–24 years group

^d^Includes people with disabilities and chronic conditions

##### Findings across age ranges

The present systematic review showed PA was strongly and negatively correlated with depression and strongly and positively correlated with general well-being in all age groups. However, different correlations between PA and negative responses occurred in different age groups. In the 6–12-year-old group, PA was not associated with stress, but was strongly and negatively correlated with anxiety and mental health problems. In the 13–18-year-old group, PA was strongly and negatively correlated with anxiety, stress, insomnia, and fatigue. In the 19–24-year-old group, findings on PA’s associations with anxiety, stress, and insomnia were inconsistent. Furthermore, PA was negatively correlated with fatigue though evidence was insufficient in the 13–18- and 19–24-year-old groups. Among the positive responses, PA was strongly and positively correlated with vigor in the 13–18-year-old group, and positively correlated in the 6–12- and 19–24-year-old groups though the evidence was insufficient. As with negative responses, we found that the 13–18-year-old group demonstrated the greatest variety of positive responses, indicating that researchers were most concerned about the responses of this age group during the COVID-19 pandemic.

##### Findings among children and youth with disabilities or chronic conditions (DCC)

Only eight negative psychological responses and four positive ones were investigated regarding the association of PA and MH among children and youth with DCC. The synthesized results showed that PA was strongly and negatively correlated with depression and anxiety in this population group (see Table 3). For example, one included study showed that children with ADHD who engaged in less than one hour of exercise per day were more likely to exhibit increased depressive symptoms during COVID-19 [94]. However, the associations of PA with all positive psychological responses were considered insufficient.

### Meta-analytic results

#### The relationship between physical activity and negative psychological responses

**Fig. 2:**
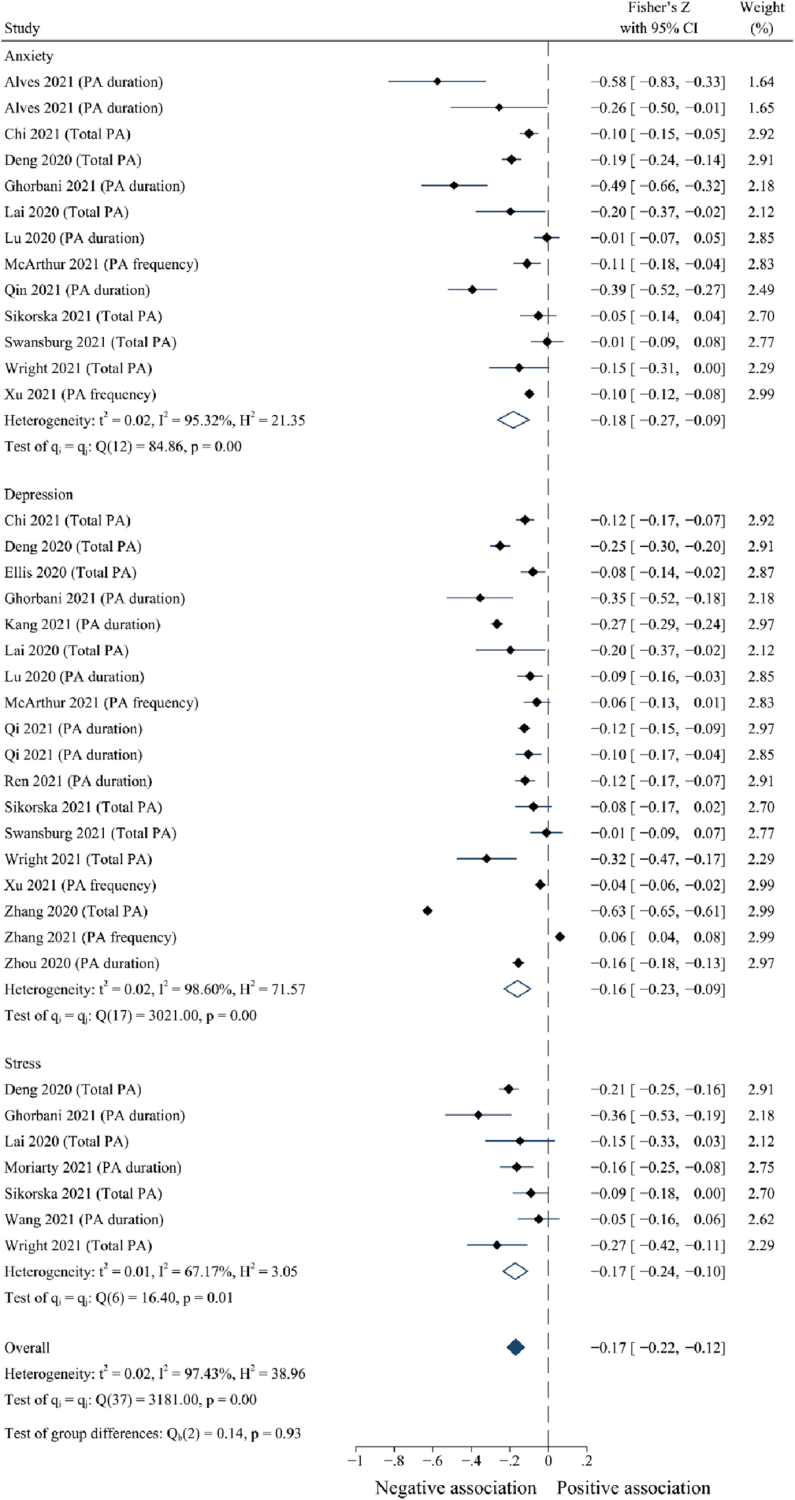
Forest plots of the relationship between physical activity and negative psychological responses during COVID-19

The meta-analysis results showed PA was weakly and negatively correlated with negative responses (Fisher’s z = − 0.170, 95% CI [− 0.22, − 0.12], *p* < 0.001, I^2^ = 92.42%) (see Fig. 2). Because Egger’s test for publication bias was significant (t = -2.50, *p* < 0.05), the trim-and-fill method was performed. After eliminating publication bias, there was a potential moderate and negative relationship between PA and negative responses (Fisher’s z = − 0.198, 95% CI [− 0.25, − 0.15], *p* < 0.001). Specifically, PA showed significant and negative associations with anxiety (Fisher’s z = − 0.180, 95% CI [− 0.27, − 0.09]; *p* < 0.001), depression (Fisher’s z = − 0.160, 95% CI [− 0.23, − 0.09], *p* < 0.001), and stress (Fisher’s z = − 0.170, 95% CI [− 0.24, − 0.10], *p* < 0.001) (see Fig. 2).

Furthermore, the meta-regression analyses results showed that age (*p* = 0.001), report of total (*p* = 0.001) and specific dimensions (duration and intensity) of PA (*p* = 0.001), and study quality (*p* = 0.001) were the primary origins of heterogeneity in the included studies for the relationship between PA and negative responses (see Table 4).

**Table 4:** Meta-regression analyses on influencing factors for the heterogeneity of included studies

Studies on negative psychological responses	β	t	P	95% CI	
Age^a^	− 0.010	− 6.52	0.001	− 0.013	− 0.007
Total PA	− 0.171	− 3.77	0.001	− 0.263	− 0.079
PA duration	− 0.219	− 4.51	0.001	− 0.319	− 0.121
PA frequency	− 0.050	− 0.50	0.622	− 0.253	0.153
PA intensity	− 0.326	− 5.49	0.001	− 0.446	− 0.206
Quality	− 0.172	− 7.04	0.001	− 0.222	− 0.123
Disability	− 0.147	− 1.99	0.053	− 0.296	− 0.002

Studies on positive psychological responses	β	t	P	95% CI	
Age^a^	0.012	4.02	0.002	0.005	0.019
Total PA	0.165	2.54	0.028	0.022	0.308
PA duration	0.090	0.40	0.697	− 0.407	0.588
PA frequency	0.202	1.78	0.103	− 0.048	0.452
PA intensity	0.350	1.71	0.115	− 0.100	0.799
Quality	0.159	3.20	0.008	0.050	0.268
Disability	0.288	1.18	0.263	− 0.249	0.824

*β* regression coefficient, *CI* confidence interval, *PA* physical activity, *t* t-statistic.

^a^Age is a continuous variable and the remaining variables are binary data.

#### The relationship between physical activity and positive psychological responses

**Fig. 3:**
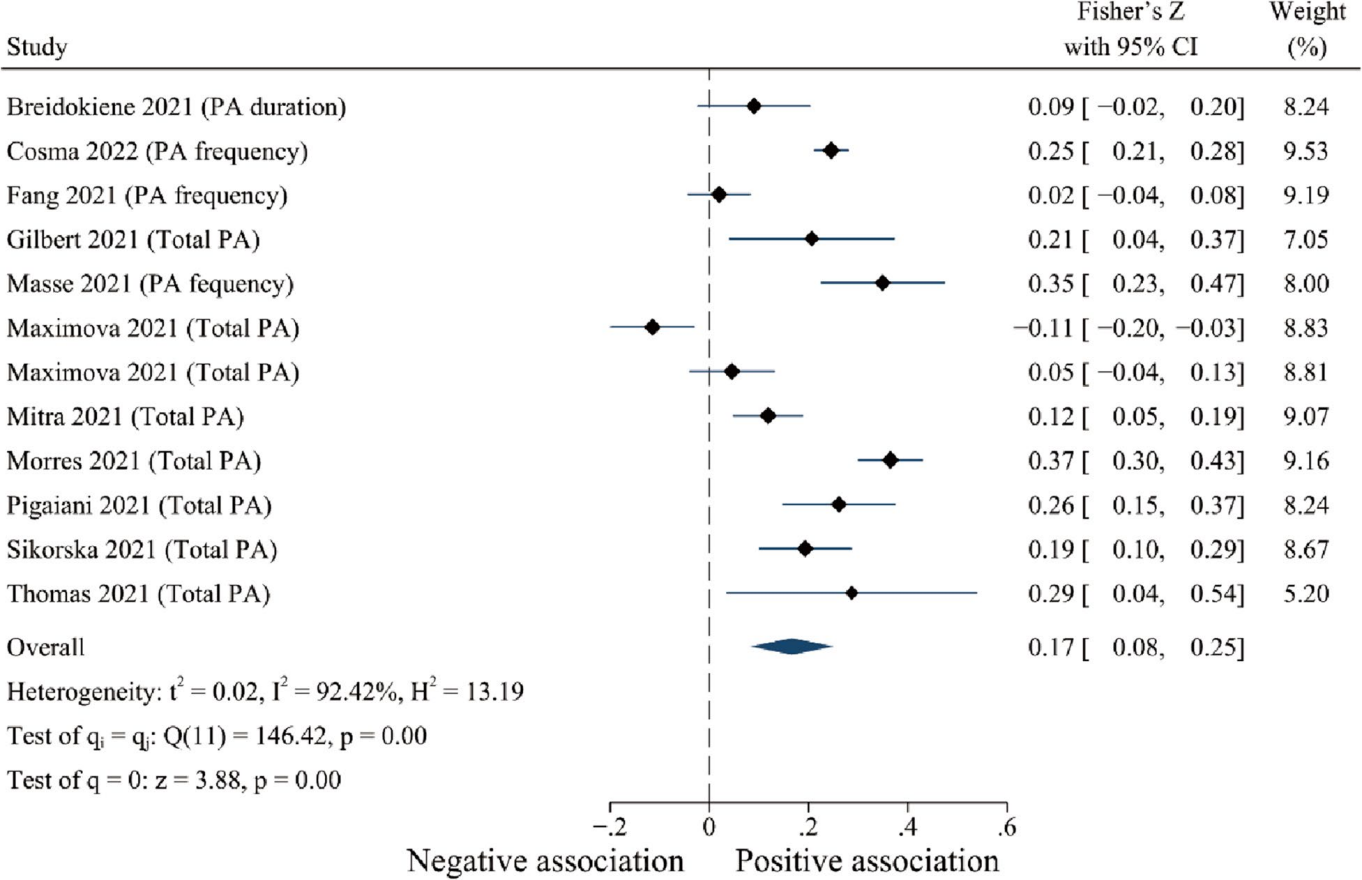
Forest plots of the relationship between physical activity and positive psychological responses during COVID-19

As illustrated in Fig. 3, PA was weakly and positively correlated with positive responses (Fisher’s z = 0.170, 95% CI [0.08, 0.25], *p* < 0.001, I^2^ = 92.42%; see Fig. 3). No significant publication bias was detected (*p* = 0.465 ). Meta-regression results showed that age (*p* = 0.002), report of total PA (*p* = 0.028), and study quality (*p* = 0.008) were the primary origins of heterogeneity in the included studies for the relationship between PA and positive responses (see Table 4).

## Discussion

This systematic review and meta-analysis examined the relationship between PA and MH among children and youth during the COVID-19 pandemic. The impacts of age group and other factors on this relationship are further discussed.

### An overview of physical activity and mental health among children and youth during COVID-19

During COVID-19, children and youth worldwide demonstrated a decreased level in PA participation that was significantly larger than before COVID-19 (36–61% of US children and youth showed declining PA levels, 50% in Ireland, 39% in Poland, 39% in Finland, 38% in The Czech Republic, and 33% in China) [39, 68, 84, 92, 109, 122–126]. Additional studies showed that only 3.6% of children (5–11 years) and 2.6% of youth (12–17 years) in Canada participated in at least 60 min of moderate to vigorous PA per day, with 49–64% of children and youth spending less PA time [80, 83, 94, 114, 127]. Another study showed that 18.9% fewer youth in Hungary engaged in moderate-intensity PA [78]. People with disabilities were also at risk for low levels of PA due to a lack of opportunities, physical education, and interventions [128]. We found studies in some countries that reported the same or even increased levels of PA among children and youth during COVID-19 compared to pre COVID-19 [102, 129–131]. However, in view of the prevalence of physical inactivity among children and youth before COVID-19 [132], PA deterioration during the pandemic and its potential health consequences deserve more attention [13].

Although COVID-19 has a significant negative impact on MH among children and youth globally, the extent of this impact varies slightly by region. For example, evidence has shown that 74% of American parents reported that their child’s MH deteriorated during the COVID-19 restriction period, and that the MH-related emergency department visiting rate of American children and youth increased by 7% [68, 133]. Similarly, it has been reported that youth with poor MH in China increased by 12% [89, 109], while in Canada, 31–44% of children felt their overall MH had declined [80, 83]. This MH deterioration among children and youth may have resulted from alienation due to physical isolation, exposure to negative news, fear that they or their loved ones would be infected by the virus, and even fear of death [30, 124].

### The relationship between physical activity and negative psychological responses

The present systematic review found that depression, anxiety, stress, insomnia, fatigue, and mental health problems were the most common negative response variables investigated during COVID-19, and all of them were significantly and negatively correlated with PA. Specifically, increases in depression and anxiety were closely related to the lack of PA, which is consistent with findings before COVID-19 [16, 17, 33]. Our meta-analysis results showed a weak and negative relationship between PA and negative psychological responses during COVID-19 (Fisher’s z = − 0.198), a finding in line with a meta-analysis published in 2019 that reported the beneficial effects of PA on negative psychological responses among children and youth [16]. Before COVID-19, the lack of PA among children and youth might have been compensated to some extent by daily commuting and school activities [33, 134]. However, COVID-19-related lockdowns and school closures resulted in at-home/indoor physical inactivity, fear, and loneliness among children and youth that ultimately amplified the impact of PA on MH [28], which in turn may have led to a further loss of interest in PA [33, 135, 136].

Additionally, our results of variable categorization showed that the correlations of PA with anxiety, stress, and insomnia among children and youth during COVID-19 was mixed in the 19–24-year-old group rather than other age groups. One reason for this may be due to that the 19–24-year-olds may have better emotional self-management ability (e.g., managing stress) with a relatively matured cognitive control system being developed in this older group than minors [137, 138]. Another possible reason is that the influencing factors on MH in the 19–24-year-old group could be more complex than that in other age ranges, thus the impact of PA on the above negative psychological responses might be diminished [139]. These findings indicate that COVID-19 not only deepened the link between PA and negative psychological responses, but also led to diverse negative response symptoms among children and youth.

COVID-19 engendered a plethora of uncommon negative psychological responses, including COVID-19 stress, fatigue, mental health problems, and negative affect [58, 61, 68, 76, 90, 105]. COVID-19 stress, as a new negative psychological response that emerged during the pandemic [65], may have been for most youth an adaptive response that prompted them to take precautions (e.g., frequent hand washing and social distancing) to protect themselves from the virus. However, some youth almost certainly experienced excessive COVID-19 stress [65], which, if not taken seriously, may have developed into post-traumatic stress when the outbreak subsided [140]. This problem, unlike depression and anxiety, can be easily overlooked and cause serious consequences to children and youth [61, 76, 89, 105, 141]. Thus, future research should pay more attention to the causes of negative psychological responses and explore further how to prevent and treat them.

Another finding from our review is that, during COVID-19, PA was significantly and negatively associated with depression, regardless of age group. We argue that school closures and physical alienation increased loneliness and decreased PA for all children and youth, which resulted in the further prevalence of depression and indirectly strengthened the connection between PA and MH [61, 112]. In addition, we found that the relationships between PA and anxiety, stress, and insomnia varied across age ranges but were strongest among 13–18-year-olds. One possible explanation is that this age group followed news about COVID-19 via social media more frequently than the other groups, which may have negatively impacted their MH [136]. Furthermore, students between 13 and 18 years of age are generally under considerable academic pressure, which may contribute further to anxiety, stress, and insomnia [61, 89, 95, 109, 142]. We also found that 13–18-year-olds presented the greatest variety of negative psychological responses during the COVID-19 pandemic. One possible reason for this was the disruption of routine due to the long-term absence of a structured school framework, which is a more important adaptation mechanism for this age group than for others [143]. The above results suggest that 13–18-year-olds were most at risk for MH problems during COVID-19. Thus, researchers need to continuously monitor members of this group since the negative psychological responses triggered by COVID-19 (e.g., COVID-19 stress and COVID-19 fear) may become long-term post-traumatic stress responses persistently affecting their MH in the post-pandemic period [144].

After experiencing COVID-19, children and youth should be informed about the prevalence of negative psychological responses during adversity, how to minimize their impact, and how to quickly achieve “closure” and move on from them [40]. Thus, instead of focusing primarily on negative psychological responses, children and youth should learn to view the loss and pain of a difficult situation with detached values.

### The relationship between physical activity and positive psychological responses

Our results demonstrated a weak and positive correlation between PA and positive psychological responses during COVID-19 (Fisher’s z = 0.17). Results of variable categorization also showed that PA was positively correlated with positive psychological responses. Specifically, PA was significantly and positively correlated with general well-being and vigor. For youth, sufficient PA can relieve psychological stress, increase mental stability, and further enhance general well-being and vigor [56]. For children, general well-being is more likely to be influenced by factors other than PA, including family cohesion, social connections with peers, and parents’ emotional states [143]. Given that fewer studies have addressed positive, compared to negative, psychological responses during COVID-19, it seems that the important relationship between PA and positive psychological responses has been to some extent overlooked. The few studies that examined this issue have demonstrated that positive psychological responses are not only closely related to PA, but also help to alleviate negative emotions [101, 145]. Thus, future research should investigate further the relationships between PA and positive psychological responses among children and youth.

### The relationship between physical activity and mental health among children and youth with disabilities or chronic conditions (DCC)

In this review, eight negative and four positive psychological response outcomes were investigated for children and youth with DCC. Of the 12 outcomes, a significant relationship between PA and MH was evident in only two negative psychological responses (i.e., anxiety and depression). The rest lacked sufficient evidence to determine a relationship with PA. Research has suggested that, due to their failure during COVID-19 to receive adequate health screenings and usual interventions, children and youth with DCC experienced more negative MH effects than their peers without DCC [56, 146]. Moreover, individuals with DCC appear to have more barriers that impede their accrual of sufficient PA, which might cause deterioration of their MH status [57, 76, 88, 147]. Although previous studies have indicated that the pandemic’s negative impacts on PA and MH were more acute for children and youth with DCC than those without [88, 108], the exact relationship between PA and MH among children and youth with DCC cannot be determined due to the limited number of studies focusing on this population group in our systematic review. Therefore, in the post COVID-19 era, researchers should focus more on PA and MH issues of children and youth with DCC.

### Limitations and recommendations for future research

Because most of the studies included in this review focused on typically developing children and youth, a meta-analysis on the relationship between PA and MH among children and youth with DCC could not be performed. Therefore, more studies on individuals with DCC should be conducted when investigating the relationship between PA and MH among children and youth. Furthermore, this review shows that studies did not include details of PA could have an impact on the relationship between PA and MH. Future studies should report PA details, such as total amount, intensity, frequency, and duration, as much as possible to avoid a one-dimensional assessment of PA.

We only included studies published up until December 2021, it was thus impossible to include research that have been conducted in the first two years of the outbreak but not yet published by the end of 2021. It is worth noting that this review included few studies with high methodological quality, which may impact the robustness of our findings. Furthermore, as only a limited number of experimental studies were eligible for this review, our meta-analyses on the magnitude of the relationship between PA and MH outcomes were based primarily on findings reported in observational studies. Thus, a cause-effect relationship between PA and MH could not be determined. Future research investigating the relationship between PA and MH among children and youth should include more studies with an experimental design and high methodological quality.

Finally, our review found that age impacted the relationship between MH and PA during COVID-19. Specifically, the most common MH problems are not the same at different ages. Future research should focus on age-specific MH problems (e.g., anxiety, stress, insomnia, and fatigue) and not limit their studies to the most common negative psychological responses, such as depression. It is important to note that limited research was conducted on preschoolers during COVID-19, which should also be addressed in future studies. Additionally, most of the existing studies were conducted in China, The United States, and Canada. In view of the potential influence of social cultural environment on the relationship between PA and MH in children and youth, future research from more countries worldwide is warranted.

## Conclusion

The present systematic review and meta-analysis confirms a close relationship between PA and MH among children and youth in terms of both negative and positive psychological responses during the COVID-19 pandemic. Negative psychological responses received disproportionately more attention than positive ones. Specifically, PA’s associations with anxiety, depression, stress, insomnia, fatigue, mental health problems, general well-being, and vigor appeared to be stronger than its associations with other MH outcomes among children and youth. In addition, the pattern and strength of relations between PA and MH outcomes varied across age ranges and health conditions, with preschoolers and those with DCC receiving less attention in the existing research. Given the importance of PA and MH for healthy development of children and youth, future research with high methodological quality should focus on (1) age-range specific relationships between PA and MH outcomes from a comprehensive perspective during the post COVID-19 era, and (2) children and youth with DCC.

## Supplementary Information

**Additional file 1: Table S1.** Details of search strategy

## Data Availability

All data are included in the manuscript and additional file.

## Abbreviations

COVID‐19: The coronavirus disease
PA: Physical activity
MH: Mental health
RCTs: Randomized controlled trials
WHO: World health organization
DCC: Disabilities or chronic conditions
