# Supplementary material for: Physical activity and mental health in children and youth during COVID-19: a systematic review and meta-analysis
Source: Child Adolesc Psychiatry Ment Health. 2023 Jul 19;17:92. doi: 10.1186/s13034-023-00629-4 (PMC10357657; doi:10.1186/s13034-023-00629-4)
Supplement: Supplementary file 1 — Additional file 1: Table S1. Details of search strategy [file 13034_2023_629_MOESM1_ESM.docx]

**Supplementary Information**

Table S1. Details of search strategy

| **Database）** | **Search Terms** |
| --- | --- |
| PubMed | All Fields =("mental health"[MeSH Terms] OR ("mental"[All Fields] AND "health"[All Fields]) OR "mental health"[All Fields] OR (("mental"[All Fields] OR "mentalities"[All Fields] OR "mentality"[All Fields] OR "mentalization"[MeSH Terms] OR "mentalization"[All Fields] OR "mentalizing"[All Fields] OR "mentalize"[All Fields] OR "mentalized"[All Fields] OR "mentally"[All Fields]) AND ("problem"[All Fields] OR "problem s"[All Fields] OR "problems"[All Fields])) OR ("mental disorders"[MeSH Terms] OR ("mental"[All Fields] AND "disorders"[All Fields]) OR "mental disorders"[All Fields] OR ("mental"[All Fields] AND "illness"[All Fields]) OR "mental illness"[All Fields]) OR ("mental disorders"[MeSH Terms] OR ("mental"[All Fields] AND "disorders"[All Fields]) OR "mental disorders"[All Fields] OR ("mental"[All Fields] AND "disorder"[All Fields]) OR "mental disorder"[All Fields]) OR ("health"[MeSH Terms] OR "health"[All Fields] OR "well"[All Fields] OR "well being"[All Fields]) OR "depress*"[All Fields] OR "anxiet*"[All Fields] OR ("stress"[All Fields] OR "stressed"[All Fields] OR "stresses"[All Fields] OR "stressful"[All Fields] OR "stressfulness"[All Fields] OR "stressing"[All Fields]) OR "happ*"[All Fields]) AND ((("physical examination"[MeSH Terms] OR ("physical"[All Fields] AND "examination"[All Fields]) OR "physical examination"[All Fields] OR "physical"[All Fields] OR "physically"[All Fields] OR "physicals"[All Fields]) AND "activ*"[All Fields]) OR "exercis*"[All Fields] OR ("sport s"[All Fields] OR "sports"[MeSH Terms] OR "sports"[All Fields] OR "sport"[All Fields] OR "sporting"[All Fields])) AND ("child"[MeSH Terms] OR "child"[All Fields] OR "children"[All Fields] OR "child s"[All Fields] OR "children s"[All Fields] OR "childrens"[All Fields] OR "childs"[All Fields] OR ("adolescences"[All Fields] OR "adolescency"[All Fields] OR "adolescent"[MeSH Terms] OR "adolescent"[All Fields] OR "adolescence"[All Fields] OR "adolescents"[All Fields] OR "adolescent s"[All Fields]) OR ("adolescent"[MeSH Terms] OR "adolescent"[All Fields] OR "youth"[All Fields] OR "youths"[All Fields] OR "youth s"[All Fields])) AND ("covid 19"[All Fields] OR "covid 19"[MeSH Terms] OR "covid 19 vaccines"[All Fields] OR "covid 19 vaccines"[MeSH Terms] OR "covid 19 serotherapy"[All Fields] OR "covid 19 serotherapy"[Supplementary Concept] OR "covid 19 nucleic acid testing"[All Fields] OR "covid 19 nucleic acid testing"[MeSH Terms] OR "covid 19 serological testing"[All Fields] OR "covid 19 serological testing"[MeSH Terms] OR "covid 19 testing"[All Fields] OR "covid 19 testing"[MeSH Terms] OR "sars cov 2"[All Fields] OR "sars cov 2"[MeSH Terms] OR "severe acute respiratory syndrome coronavirus 2"[All Fields] OR "ncov"[All Fields] OR "2019 ncov"[All Fields] OR (("coronavirus"[MeSH Terms] OR "coronavirus"[All Fields] OR "cov"[All Fields]) AND 2019/11/01:3000/12/31[Date - Publication]) OR ("pandemic s"[All Fields] OR "pandemically"[All Fields] OR "pandemicity"[All Fields] OR "pandemics"[MeSH Terms] OR "pandemics"[All Fields] OR "pandemic"[All Fields]) OR ("sars cov 2"[MeSH Terms] OR "sars cov 2"[All Fields] OR "sars cov 2"[All Fields]))  Filters: English, Humans |
| Web of Science | TS: Topic  TS =((mental health OR mental problem OR mental illness OR mental disorder OR well-being OR depress* OR anxiet* OR stress OR happ*) AND (physical activ* OR exercis* OR sport) AND (child OR adolescent OR youth) AND (COVID-19 OR pandemic OR SARS-CoV-2))  Filters: English, Article |
| Embase | Quick Search =('mental health'/exp OR 'mental health' OR (mental AND ('health'/exp OR health)) OR 'mental problem' OR (mental AND problem) OR 'mental illness'/exp OR 'mental illness' OR (mental AND ('illness'/exp OR illness)) OR 'mental disorder'/exp OR 'mental disorder' OR (mental AND ('disorder'/exp OR disorder)) OR 'well being'/exp OR 'well being' OR depress* OR anxiet* OR 'stress'/exp OR stress OR happ*) AND (physical AND activ* OR exercis* OR 'sport'/exp OR sport) AND ('child'/exp OR child OR 'adolescent'/exp OR adolescent OR 'youth'/exp OR youth) AND ('covid 19'/exp OR 'covid 19' OR 'pandemic'/exp OR pandemic OR 'sars cov 2'/exp OR 'sars cov 2') |
| APA PsycInfo | All Fields =(mental health OR mental problem OR mental illness OR mental disorder OR well-being OR depress* OR anxiet* OR stress OR happ*) AND (physical activ* OR exercis* OR sport) AND (child OR adolescent OR youth) AND (COVID-19 OR pandemic OR SARS-CoV-2) |
